# Supplementary material for: Hazard Assessment of the Effects of Acute and Chronic Exposure to Permethrin, Copper Hydroxide, Acephate, and Validamycin Nanopesticides on the Physiology of Drosophila: Novel Insights into the Cellular Internalization and Biological Effects
Source: Int J Mol Sci. 2022 Aug 14;23(16):9121. doi: 10.3390/ijms23169121 (PMC9408976; doi:10.3390/ijms23169121)
Supplement: Supplementary file 1 [file ijms-23-09121-s001.zip › ijms-1849090-supplementary.pdf]

Supplementary Figure captions

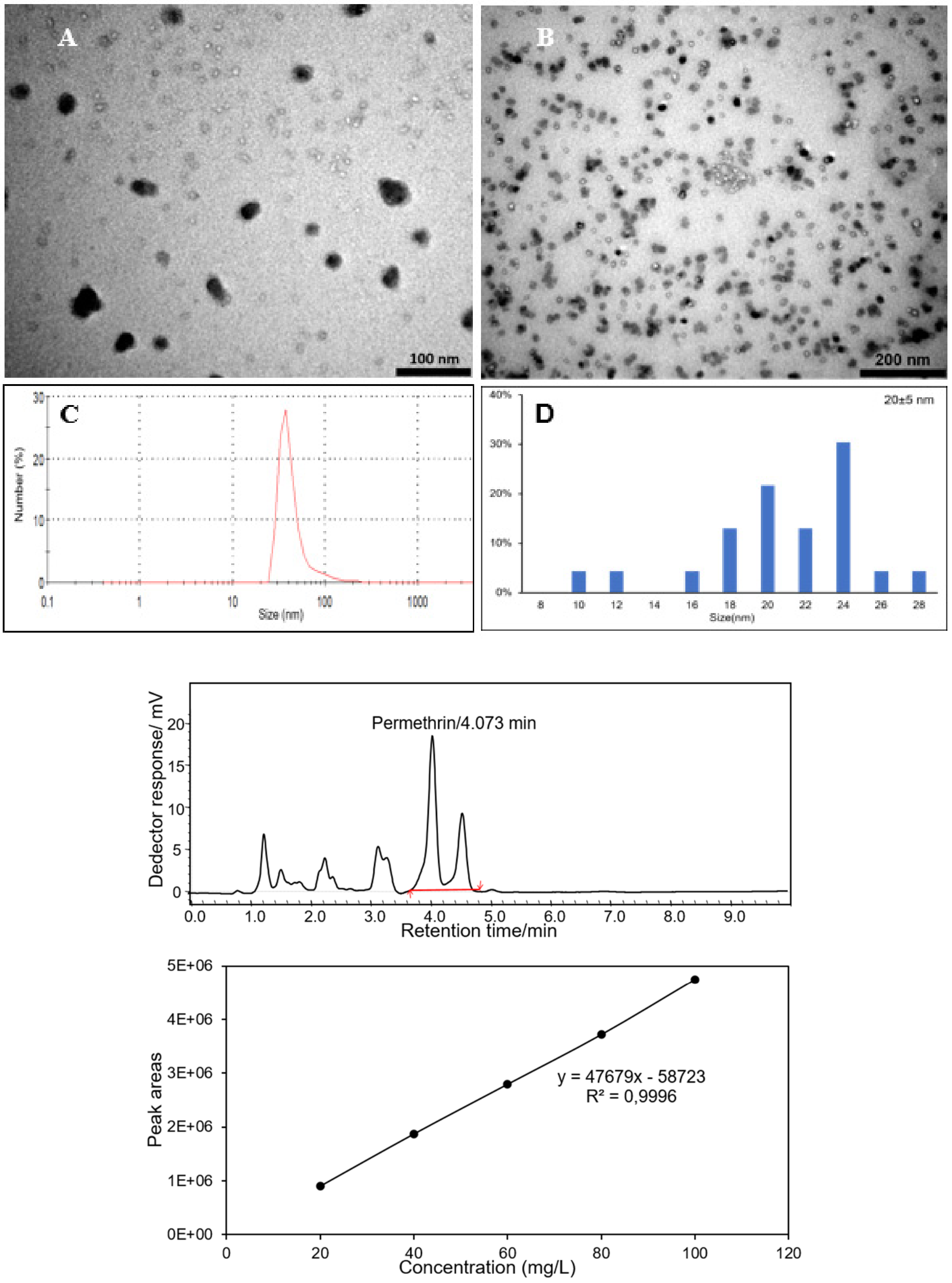

E

Figure S1. Characterization of permethrin nanopesticides (20 ± 5 nm). (A-B) Typical TEM images. (C) represents size distribution by DLS characterization. (D) size distribution histogram using such images. (E) HPLC spectrum and calibration curve of permethrin.

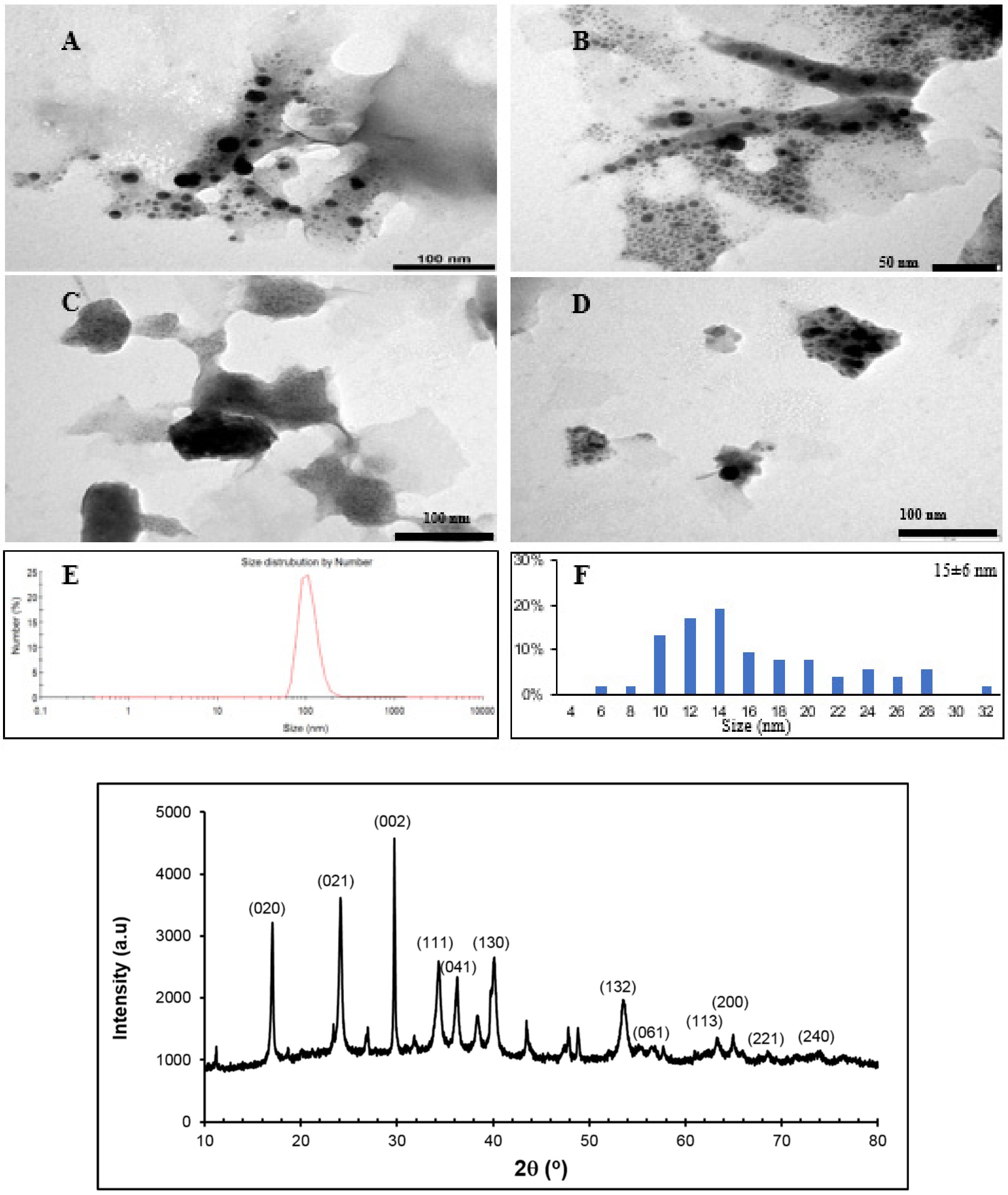

G

Figure S2. Characterization of $Cu(OH)_2$ nanopesticides (15 ± 6 nm). (A-D) Typical TEM images. (E) represents size distribution by DLS characterization. (F) size distribution histogram using such images. (G) XRD diffraction pattern.

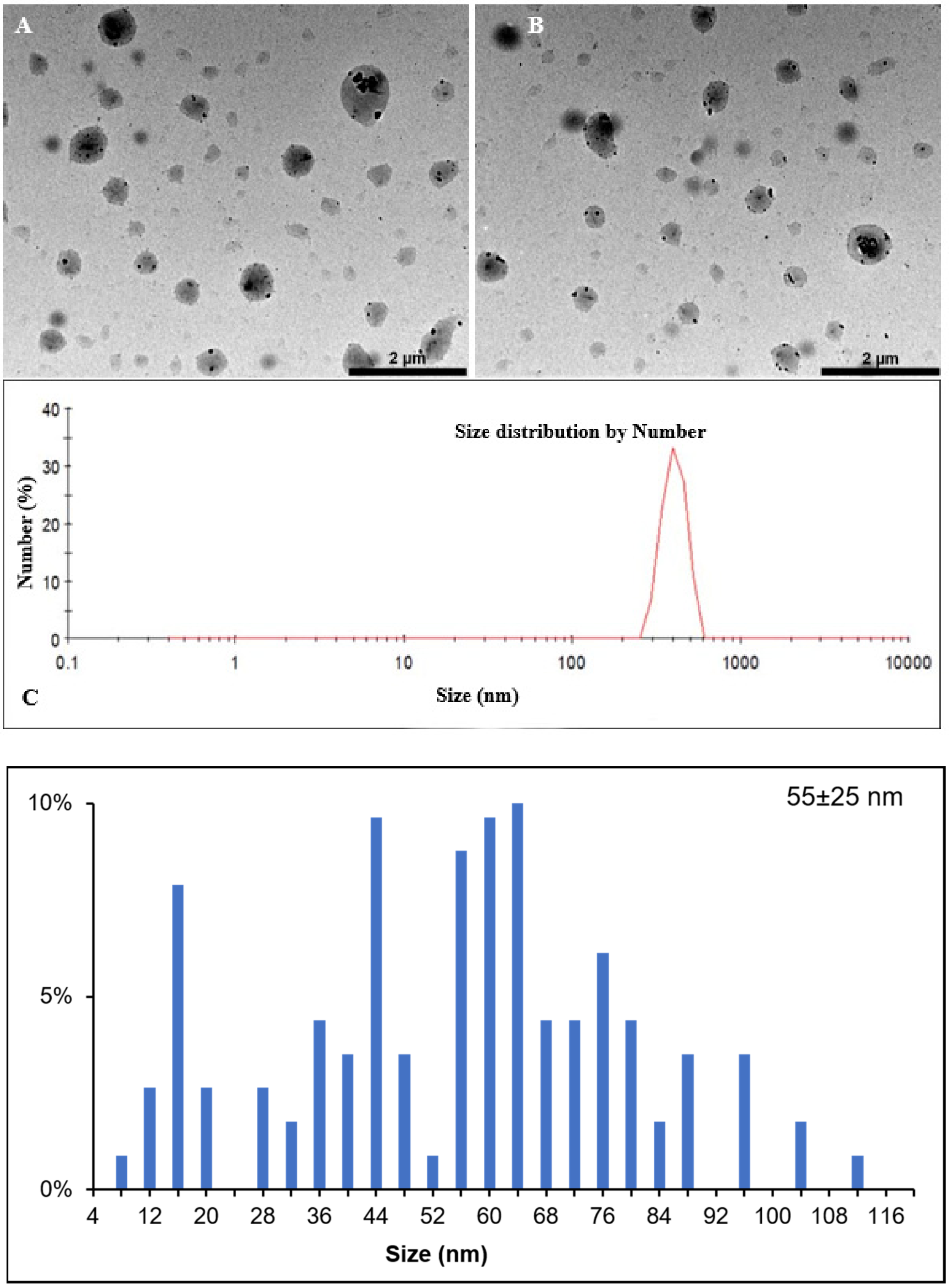

D

Figure S3. Characterization of acephate nanopesticides (55 ± 25 nm). (A-B) Typical TEM images. (C) represents size distribution by DLS characterization. (D) size distribution histogram using such images.

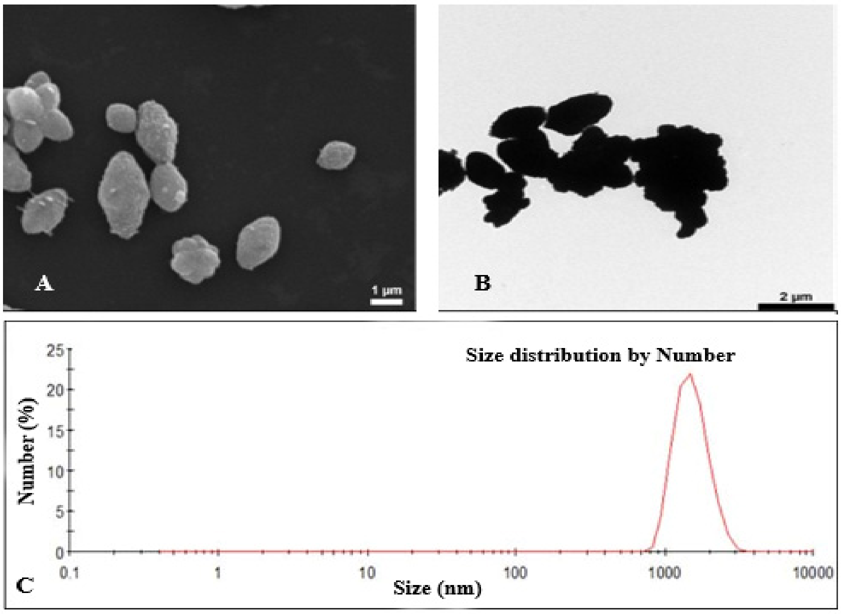

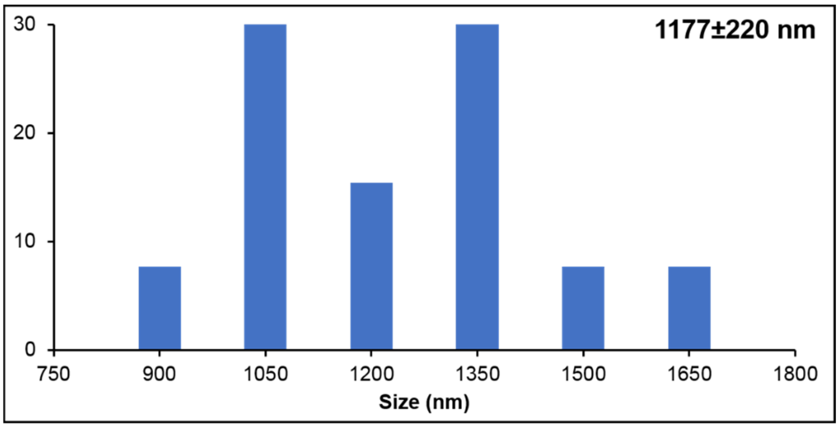

D

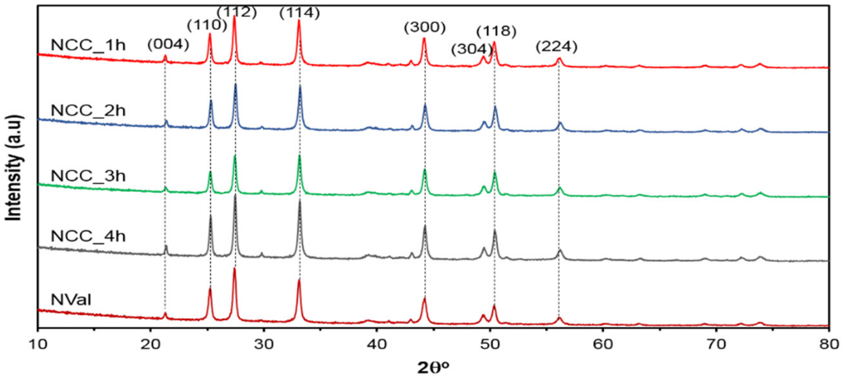

E

Figure S4. Characterization of validamycin nanopesticides (1177 ± 220 nm). (A) Typical SEM image. (B) typical TEM image. (C) represents size distribution by DLS characterization. (D) size distribution histogram using such images. (E) XRD diffraction pattern.

A.

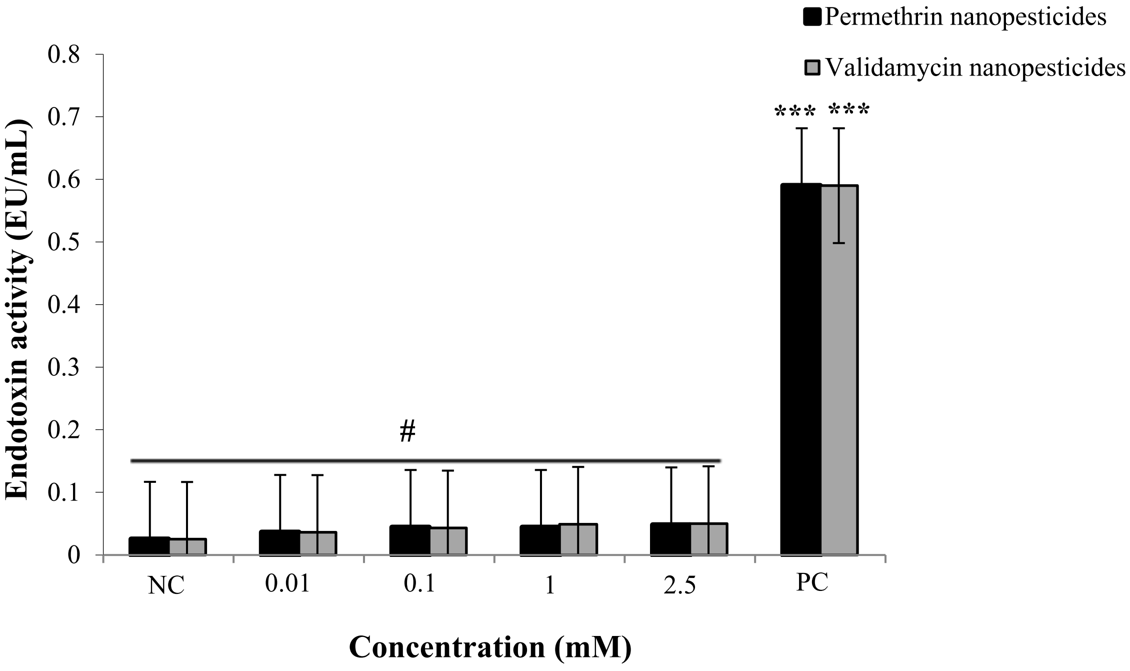

B.

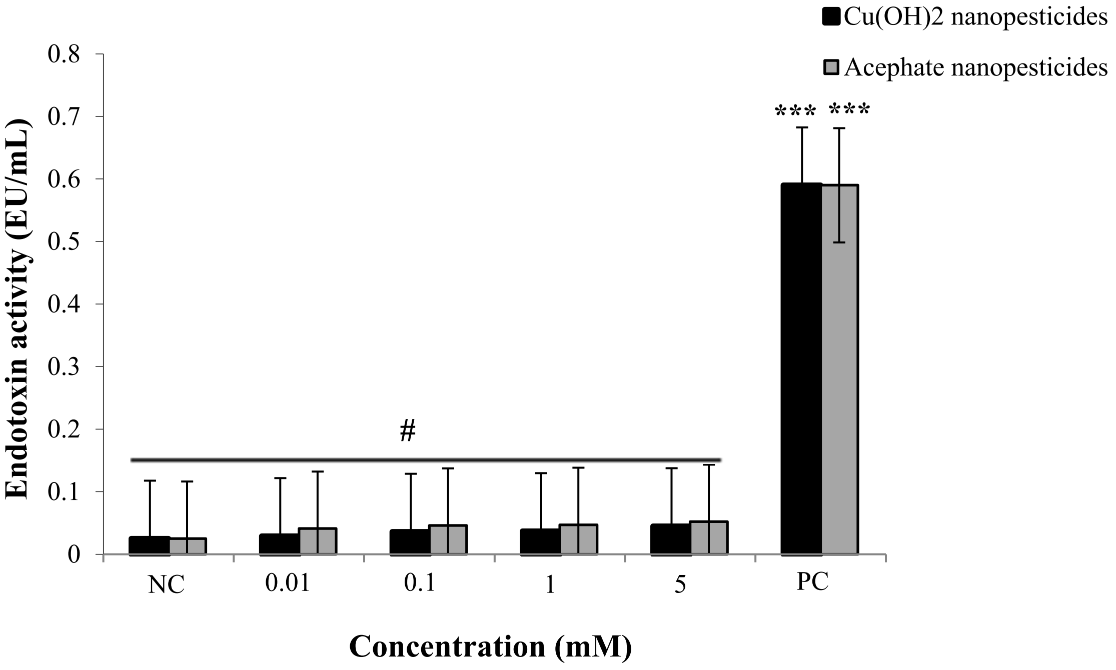

Figure S5. Endotoxin level (EU/mL) in different concentrations of permethrin nanopesticides and validamycin nanopesticides (A) and $Cu(OH)_2$ nanopesticides and acephate nanopesticides

(B) detected using chromogenic Limulus amebocyte lysate (LAL) assay. Each point represents a mean of 3 replicates ± standard error. Assays were performed according to the kit protocol (Lonza, QCL-1000™). Lipopolysaccharide (LPS) (0.5 EU/ml) was used as the positive control (PC). # The endotoxin level of the nanopesticides was below the limit of detection (0.116667 EU/ml). $^{***}P \leq 0.001$ when compared with the negative control (LAL reagent water) using the Student's *t*-test.

Supplementary Tables

Table S1. Summary of the mortality rates and LC_{50} values obtained in the *Drosophila* larvae after exposure to permethrin and permethrin nanopesticides.

Test compounds	Concentration (mM)	Mortality rate (± SE)	LC_{50} values (95% lower and upper confidence limits)	Slope (± SE)
	Distilled water	0 ± 0.0		
	Ethanol (2%)	6 ± 1.3 a		
	Nano-capsule (2.5 mM)	7 ± 1.1 a		
Permethrin	0.01	8 ± 1.5 a	0.1 (0.08-0.45)	9.108 (0.32)
	0.03	16 ± 3.1 a		
	0.06	27 ± 3.6 b		
	0.1	48 ± 7.2 c		
	0.5	67 ± 3.1 d		
	1	71 ± 3.9 d		
	2.5	76 ± 2.6 d		
	5	80 ± 5.2 e		
	7.5	83 ± 4.7 e		
	10	87 ± 3.1 e		
Permethrin nanopesticides	0.01	9 ± 1.2 a	2.5 (1.38-4.13)	9.272 (0.37)
	0.03	12 ± 2.8 a		
	0.06	18 ± 3.1 b		
	0.1	22 ± 6.4 b		
	0.5	26 ± 2.6 b		
	1	32 ± 3.2 c		
	2.5	49 ± 2.9 d		
	5	78 ± 5.6 e		
	7.5	84 ± 4.2 e		
	10	90 ± 2.2 e		

SE: Standard Error

Means within a column followed by the same lower letter are not significantly different as per Duncan Multiple Range test ($P \leq 0.05$)

Table S2. Summary of the mortality rates and LC_{50} values obtained in the *Drosophila* larvae after exposure to $CuSO_4.5H_2O$ and $Cu(OH)_2$ nanopesticides.

Test compounds	Concentration (mM)	Mortality rate (± SE)	LC_{50} values (95% lower and upper confidence limits)	Slope (± SE)
	Distilled water	0 ± 0.0		
	0.01	6 ± 1.7 a		

$CuSO_4.5H_2O$	0.1	18 ± 2.7 a	5 (2.91-5.83)	10.46 (0.67)
	0.5	21 ± 3.2 b		
	1	27 ± 6.1 b		
	2.5	31 ± 3.3 b		
	5	48 ± 3.5 c		
	7.5	72 ± 2.2 d		
	10	90 ± 5.7 e		
$Cu(OH)_2$ nanopesticides	0.01	5 ± 1.5 a	5 (2.93-5.47)	10.48 (0.59)
	0.1	10 ± 2.4 a		
	0.5	16 ± 2.5 a		
	1	22 ± 6.1 b		
	2.5	28 ± 3.2 b		
	5	49 ± 3.6 c		
	7.5	70 ± 2.6 d		
	10	86 ± 5.2 e		

SE: Standard Error

Means within a column followed by the same lower letter are not significantly different as per Duncan Multiple Range test ($P \leq 0.05$)

Table S3. Summary of the mortality rates and LC_{50} values obtained in the *Drosophila* larvae after exposure to acephate and acephate nanopesticides.

Test compounds	Concentration (mM)	Mortality rate (± SE)	LC_{50} values (95% lower and upper confidence limits)	Slope (± SE)
	Distilled water	0 ± 0.0		
	Nano-capsule (PEG-400) (5 mM)	6 ± 1.7 a		
Acephate	0.01	7 ± 2.1 a	5 (2.78-6.43)	10.8 (0.83)
	0.1	10 ± 2.9 a		
	0.5	22 ± 3.5 b		
	1	30 ± 5.3 b		
	2.5	35 ± 2.9 b		
	5	49 ± 3.2 c		
	7.5	78 ± 2.7 d		
	10	86 ± 4.4 e		
Acephate nanopesticides	0.01	4 ±1.1 a	5 (2.86-6.32)	10.36 (0.91)
	0.1	8 ± 2.2 a		
	0.5	18 ± 3.1 a		
	1	27 ± 6.6 b		
	2.5	32 ± 2.8 b		
	5	48 ± 3.9 c		
	7.5	72 ± 2.1 d		
	10	81 ± 4.8 e		

SE: Standard Error

Means within a column followed by the same lower letter are not significantly different as per Duncan Multiple Range test ($P \leq 0.05$)

Table S4. Summary of the mortality rates and LC_{50} values obtained in the *Drosophila* larvae after exposure to validamycin and validamycin nanopesticides.

Test compounds	Concentration (mM)	Mortality rate (± SE)	LC_{50} values (95% lower and upper confidence limits)	Slope (± SE)
	Distilled water	0 ± 0.0		
	Nano-capsule (2.5 mM)	6 ± 2.8 a		
$CaCO_3$	0.01	4 ± 3.3 a	5 (2.77-5.92)	10.86 (0.77)
	0.1	16 ± 2.2 b		
	0.5	27 ± 4.1 c		
	1	35 ± 5.9 c		
	2.5	39 ± 2.2 d		
	5	48 ± 3.5 e		
	7.5	80 ± 2.1 f		
	10	87 ±3.7 f		
Validamycin	0.01	8 ± 2.1 a	2.5 (1.82-5.08)	11.33 (0.65)
	0.1	15 ± 1.8 a		
	0.5	24 ± 1.9 b		
	1	32 ± 3.7 b		
	2.5	49 ± 2.6 c		
	5	67 ± 3.1 d		
	7.5	77 ± 3.3 d		
	10	86 ± 3.8 e		
Validamycin nanopesticides	0.01	3 ± 1.6 a	2.5 (1.95-4.47)	11.26 (0.53)
	0.1	11 ± 2.5 a		
	0.5	19 ± 2.4 b		
	1	30 ± 4.2 b		
	2.5	47 ± 2.1 c		
	5	64 ± 3.3 d		
	7.5	73 ± 3.7 d		
	10	83 ± 4.2 e		

SE: Standard Error

Means within a column followed by the same lower letter are not significantly different as per Duncan Multiple Range test ($P \leq 0.05$)
